# 2007 EORTC-NCI-ASCO Annual Meeting: Molecular Markers in Cancer

**DOI:** 10.3332/eCMS.2008.75

**Published:** 2008-02-18

**Authors:** C Lukan

**Affiliations:** Avenue E. Mounier, 83 b11, 1200 Brussels, Belgium

## Abstract

The recent EORTC-NCI-ASCO Annual Meeting on ‘Molecular Markers in Cancer’ was held on 15–17 November 2007 in Brussels, Belgium. It was the largest meeting to date and marked the first year in which the American Association of Clinical Oncology (ASCO) joined in the efforts of the European Organisation for Research and Treatment of Cancer (EORTC) and the National Cancer Institute (NCI) in organizing this annual event. More than 300 clinicians, pathologists, laboratory scientists and representatives from regulatory agencies and the pharmaceutical industry came together for three days of intense discussion, debate and reflection on the latest biomarker therapeutic discoveries, strategies and clinical applications. The poster discussion sessions featured 79 research abstracts. The three most outstanding abstracts, all authored by young female researchers, were selected for presentation during the main meeting sessions. Highlights of each scientific session are presented.

## Opening lecture

Mr. Janez Potoènik, the EU Commissioner for Science and Research, delivered the opening lecture applauding the dedication of the EORTC over the past 45 years to the promotion and conduct of multidisciplinary cancer research. He welcomed the transatlantic partnership between the EORTC, the NCI and ASCO as an exemplary approach to the global research challenges of cancer. Speaking on the topic of European research in cancer, Commissioner Potoènik cited the development of the European Research Area that promotes the free movement and seamless interaction of researchers; reinforces the links between education, research and innovation; supports research through the coordination of national, regional and European programs and builds mutually beneficial international partnerships.

The EU investment in cancer research, through the 6th Framework Programme, spanning 2002–2006, witnessed an increase from €18 million to €450 million. The first €70 million of a similar amount has already been released under the 7th Framework Programme with an emphasis on translational research projects that will ‘exploit the European dimension by combining resources and complementary competences from several European countries, and by encouraging the comparison of results and data from the whole of Europe,’ according to Commissioner Potoènik. Additional funding has also been earmarked by the European Research Council (ERC) for a network infrastructure for biotherapy facilities; an European bio-banking and bio-molecular resources infrastructure and an European advanced translational research infrastructure.

Commissioner Potoènik urged Member States to work together in cancer research, which will be facilitated in part through the planned ERA-NET for the coordination of national research programmes and an initiative using cancer registries for research purposes. The upcoming 2008 Slovenian Conference on Cancer ‘The Burden of Cancer -- How Can Be Reduced?’ will address ways to improve the structure and coordination of cancer research in the EU, increase research funding, translate knowledge into applications and include patients in the process of cancer research.

## Assessment of biomarkers in tumour tissues and blood

The first scientific session of the meeting featured presentations highlighting the challenges researchers face working in the area of tumour marker assessment and the standards used for the collection, storage, transportation and analysis of biospecimens. Vital international efforts are ongoing at this time to further define the procedures and methods used in the area of biobanking to ensure the collection of the highest quality of biospecimens and to validate the outcomes of this research. It is clear that greater international harmonization is needed in this respect.

The newly updated ASCO recommendations for the use of the tumour marker tests in the prevention, screening, treatment and surveillance of breast cancer is one such effort. The 2007 update includes six new tumour marker categories that are now recommended for use in practice based on clinical utility and magnitude of benefit. Dan Hayes, the ASCO Committee Co-Chair pointed out, however, that the routine use of other markers such as DNA/ploidy by flow cytometry, p53, cathepsin D, cyclin E, proteomics, certain multi-parameter assays, bone marrow micro-metastases detection and circulating tumour cells cannot be recommended at this time due to a lack of sufficient levels of supporting evidence.

Another set of forthcoming recommendations for the collection and handling of bio-specimens prepared by the Blood, FFPE and Fresh/Frozen Tissue Working Groups of the NCI-sponsored North American Breast Cancer Cooperative Groups and the Breast International Group were highlighted by Brian Leyland-Jones of Emory University School of Medicine. This work aims to promote and ensure the standardized collection of high-quality specimens, guarantee the application of future technologies and incorporate these standards into all future clinical trial protocols conducted by the Breast International Group (BIG) and NCI-Sponsored North American Cooperative Groups in an effort to maximize results from specimen-based diagnostic testing and research.

Cancer biobanking from both an American and European perspective was discussed. Carolyn Comptom from the National Cancer Institute, Bethesda, MD, noted that although translational research promises to advance molecular medicine for cancer patients, the single biggest current roadblock is the lack of sufficient high-quality biospecimen materials needed to study the biological complexities of genomics, proteomic and metabolomics. The Office of Biorepositories and Biospecimen Research (OBBR) at the US National Cancer Institute has undertaken several new initiatives to address these issues, including biobanking protocols, patients’ consent, biobanking workforce education and the establishment of a new field of research for biospecimen science.

Christian Chabannon (Institut Paoli-Calmettes, Marseilles, France) pointed out that in Europe the primarily hospital-based biobanking system faces numerous challenges for the collection and annotation of high-quality biological materials needed for translational research. To address these challenges, local, regional and national efforts are being made for specimen tracking and management, institutional collaboration to establish validated biospecimen collection catalogues, financial support for restructuring of biobank facilities and quality assurance standards. Current European initiatives such as the Tubafrost, EuroBioBank and European Biobanks United Facility are significant initiatives towards a more centralized coordinated approach to biobanking.

Gerry Thomas, Imperial College and Wales Cancer Bank furthered the debate elaborating on the importance of adherence to and the monitoring of quality assurance (QA) standard operating procedures (SOPs) for the collection, documentation and storage of biospecimens in the development of clinical biomarkers. Her recommendations for a system of QA include the strict use of and adherence to written SOPs. Furthermore, QA at all subsequent stages is required when modifying the methods of specimen collection. Additional requirements are the need for careful assessment of storage time and methods, the appropriate application of technology, the most suitable QA depending on the intended use of the biological material and the constant need to educate researchers. These measures help to ensure a high quality of clinical biomarker research.

The Bavarian Red Cross ‘Blood Donor Biobank,’ in operation since 2006, is an example of a well-established biobanking system supported by experienced employees and GMP facilities, SOPs and QA processes. According to Silke Martin, Head of the Blood Donor Biobank, it represents a unique opportunity and a new approach for identifying and validating tumour markers. Whereas others are only in the start-up process of establishing biobanking facilities, this biobank already has a repository of over 4 million plasma samples prospectively collected from healthy volunteers linked to donor health status. The Bavarian experience demonstrates that blood banks are ideal cancer research units owing to the ease of access, collection, transportation, storage and analysis that blood samples provide. Both academic and industry research collaborations are currently ongoing to screen for and identify potential cancer biomarkers.

The roundtable discussion addressed the importance of harmonization, funding and patient/public support for biobanking and tumour marker development. Harmonization is needed in the global standardization of biobanking activities, the process of biomarker validation and the need for improved partnerships with key stakeholders to ensure the long-term financial viability of biobanking. The global standardization of biobanking requires universal guidelines for biospecimen collection, transportation, storage and cataloging. Improved communication are needed to ensure the appropriate financial support, the availability of biobanking facilities and the adaptation of global standards by local institutions that may be achieved in part by the support of advocacy groups and the public exerting pressure on the politicians. International collaboration and harmonization will ensure the most efficient use of funding, the longevity of established facilities and the future career of young translational researchers. Biobanking is now a part of the evolution of healthcare. Currently, most cancer-related healthcare costs occur in the last 6 months of life. Biobanking and the development of biomarkers and targeted therapies that ‘select’ patients for more tailored therapeutic approaches earlier, requires upfront investment but will result, not only in a more efficient use of funds but ultimately advance the battle to prevent cancer.

## Imaging biomarkers

This session examined the current state of research on the use of imaging methods as predictive and treatment response biomarkers. Lawrence Schwartz, of Memorial Sloan-Kettering Cancer Centre, provided insight into whether size-based response criteria are appropriate in today’s era of targeted therapy. Despite the many advances in imaging technology and the use of new targeted therapies (i.e. bevacizumab, erlotinib, gefitinib), clinicians today continue to use computed tomography (CT) and assess tumour size based on the RECIST or WHO criteria. These therapeutic agents, however, induce tumour changes in response to therapy that CT may not reliably reflect. Early results indicate that volumetric CT calculation, measuring tumour central necrosis and cystic change and tumour ‘ghosting’ in response to treatment may represent future response biomarkers that complement current criteria or even replace them.

Wolfgang Weber shared the findings of work done at the University of Freiburg, evaluating the utility of FDG-PET to predict early treatment response and patient outcome based on the ability of FDG-PET to differentiate residual or recurrent viable tissue and therapy-induced fibrosis. This ability would allow clinicians to personalize patient’s therapy and reduce the adverse effects and costs of ineffective or unnecessary treatments. He suggested that FDG-PET could also reduce the length of new drug clinical trials via the provision of earlier and more accurate measures of tumour therapy response as already demonstrated by studies of the c-kit inhibitors for gastrointestinal stromal tumours. In these studies, tumour size reduction was preceded by metabolic changes. Promising signs for the future use of this technology include the recent standardization of FDG-PET imaging that now allows for its use at non-research medical centres and the current inclusion of FDG-PET response assessment in the International Working Group Criteria for lymphoma.

The PET biomarker research is also ongoing at the University of Washington, and David Mankoff discussed the use of FDG-PET as functional and molecular imaging markers for selecting patient treatment. FDG-PET has shown its value as a biomarker of treatment response in studies such as the MUNICON gastroesophageal cancer study, which showed the ability of FDG-PET to predict early treatment response based on metabolic changes. Radiopharmaceuticals can also guide treatment selection by quantifying the treatment target and by identifying treatment resistance factors. PET can measure therapeutic targets such as ER, AR and HER2 molecules in breast and prostate cancer, using fluoroestradiol (FES), F-18-fluorodihydrotestosterone (FDHT) and [Ga-68]-labelled F(9ab’) trastuzamab fragments. [F-18]-flourosionidazole PET can measure tumour hypoxia, a known resistance factor for radiotherapy. Tumour drug resistance due to blocked tumour drug delivery can be shown by using [C-11]-verapamil to measure regional P-glycoprotein transport. PET can also detect early treatment response by measuring tumour proliferation and/or cell death. New radiopharmaceuticals utilised together with conventional and established PET procedures are ideally suited for use in the early pre-clinical and phase 0-I testing of new targeted therapies but will require appropriate standards of testing and validation.

Gordon Jayson of the Christie Hospital presented an opposing view on optimal drug sequencing of combination treatments suggesting that by administering cytotoxic agents prior to anti-angiogenic agents, contrary to current opinion, improved therapeutic benefit may be observed. Dynamic contrast-enhanced MRI (DCE-MRI) has been used as a biomarker to evaluate VEGF inhibitors (vascular endothelial growth factor) based on change in tumour vascular permeability as measured by the leakage of contrast media from the tumour vessels into the interstitial space and expressed as Ktrans. Investigators at the University of Manchester and the Christie Hospital have observed that the anti-angiogenic impact of VEGF inhibitors on Ktrans may be regulated by a more widespread effect on the VEGF system then currently believed. As the use of anti-angiogenic drugs becomes more widespread, well-established DCE-MRI parameters will be needed. The importance of this technology as a ‘response biomarker’ requires its evaluation as part of future early clinical studies testing new drugs.

The roundtable discussion focused on methods to evaluate and qualify imaging biomarkers in drug development and their use as surrogate markers of clinical benefit. The need for an international conference to coordinate the standardization of biomarker imaging is recognized due to the complexity of each imaging domain. Imaging-base biomarker work is reproducible in small early phase studies however when attempting large phase III confirmative trials multiple issues arise that include not only the technique of the imaging procedure itself but also extend to the scanners used, scanner installation and the use of similar algorithms to interpret the results. Progress is being made with FDG-PET technology. However, partnership is needed with the scanner industry to stimulate further development and standardization. Another issue surrounds the use of radiopharmaceuticals as biomarker probes and the validation process. Each is a therapeutic product in itself and therefore requires phase I to III testing with subsequent approval for human use. The NCI Cancer Imaging Network currently conducts early phase imaging drug studies in this respect. The final point raised by the discussants was the need for new study designs that define valid endpoints of response when testing functional or molecular imaging. Despite the advances to date and the promising uses of imaging as biomarkers of treatment response, much work lies ahead and must be addressed by the combined and coordinated effort of all stakeholders.

## Tumour biomarker assessment at the gene and protein level

This session provided a broad view of the in-depth knowledge underlying the use of biomarkers for the diagnosis, prognosis and treatment of various types of cancers. Christos Sotiriou from the Institut Jules Bordet presented the results of the first and largest meta-analysis conducted of gene expression profiles in breast cancer. This study evaluated gene expression and clinical data from 2833 breast tumours. The analysis showed that the disparity in gene lists studied was attributed to patient heterogeneity, the methods of gene profiling and small sample size compared to examined gene number resulting in sampling variation. Tumours were classified into three main subtypes based on ER and ERBB2+ status. ER-/ERBB2- and ERBB2+ tumours were characterized by high proliferation, whereas ER+ tumours were more heterogeneous and further subdivided into ER+/low/luminal A and ER+/high/luminal B subtypes. All examined prognostic signatures, despite gene list disparity, had similar performance and the common significant factor appeared to be proliferation. All signatures proved valuable for prognosticating risk of recurrence in the ER+ subgroups and less so for the ER- and ERBB2+ tumours. The researchers concluded that breast cancer tumour size and nodal status remain important but that an association does exist between clinicopathological prognostic factors, expression-based sub-typing and prognostic signatures.

Janet Warrington, Affymetrix, Inc., presented the challenges in applying genomic technology to molecular diagnostics highlighting that despite the use of whole genome gene expression assays in cancer studies today, few diagnostic assay tests have been developed and approved for widespread use. This relates back to assay reproducibility, outcome data reliability and whether the gene signature is of any clinical relevance. A bottleneck exists in the development process of diagnostic assays at the time of clinical validation especially with respect to study design. Stefan Michiels noted, ‘the prognostic value of published microarray results in cancer studies should be considered with caution’ and recommended the use of validation by repeated random sampling. International standard setting initiatives are currently underway in an attempt to expedite the use of micro-array technology in clinical studies, trials and diagnostics. The Clinical Laboratory Standards Institute and the Microarray Quality Control (MAQC) project are attempts at standardising the process of material collection and handling, and the testing of assay reproducibility. These steps are all needed before the full benefits of genomic technology can be derived for not only researchers and developers but also ultimately for the patient.

Samir Khleif presented the FDA perspective for testing and use of biomarkers in oncology. He highlighted that ‘few biomarkers actually reach clinical use and that a new way of approaching biomarker research is urgently needed’. The word biomarker was first used in 1965. However, PSA was approved as a marker to monitor treatment in 1986 and only in 1994 was approved for early detection of prostate cancer. The challenges facing biomarker development include laboratory methodology, analytical validation, and clinical quantification, cost utility and regulatory hurdles. Clinical trial design has also fallen far behind compared to the advanced nature of technology and drugs tested today. The FDA supports a shift in the way drugs are developed, evaluated and assessed for human use, including a move away from the conduct of massive-sized clinical trials and more towards the study of specific patient subsets and personalized tailored therapies. This approach requires partnerships at all stages of drug development and is the focus of the current ‘New Federal Health Initiative to Improve Cancer Therapy’, which is an attempt to accelerate the development and delivery of new cancer treatments for patients.

The technology and tradeoffs of mining the proteome for clinically useful lung cancer signatures was explored by David Carbone of Vanderbilt University. The various classes of lung cancer each contain many distinct and overlapping subsets, all requiring specific analyses to define the optimal therapeutic approach. Patients with tumours that express mutant EGFR derive a significant benefit from minimally toxic targeted therapy such as gefitinib and erlotinib. However, further study of potential salvage therapies is required to address the eventual emergence of resistance mechanisms. Evaluation of the proteome compared to DNA sequence analysis or expression arrays has multiple advantages. A complete understanding of the proteome would provide information on the mechanism of functional dysregulation associated with the development of cancer (DNA mutations, rearrangements, transcriptional alterations and promoter methylation). In-depth information is now obtainable using the improved previously cumbersome technology of shotgun proteomics. This approach allows for the analysis of single tissue samples, and the identification of tumour cell line activated pathways in real time as well as direct quantification of specific peptides. Further refinement will permit the assessment of individualised risk and aid in the treatment decision-making process.

Elftherios Diamandis discussed novel prognostic and treatment biomarkers for patients with ovarian cancer. His group work at Mount Sinai Hospital in Toronto has focused on the human kallikrein locus that consists of 15 genes with significant similarity. They have developed methods to quantify all kallikrein proteins in serum and tissue extracts and have identified six kallikreins with prognostic value that are specifically over-expressed in ovarian cancer (KLK5, KLK6, KLK8, KLK10, KLK11, KLK13). Through an elaborate analysis of these kallikreins, alone and in combination with other biomarkers (CA-125) and clinical parameters, they have succeeded in developing a combined marker that is both prognostic and predictive of ovarian cancer patient response to chemotherapy. High serum levels of KLK6 were associated late stage disease, lack of response to chemotherapy and poor disease-free and overall survival. KLK6 values were 49 times higher when compared to normal ovarian tissue. The use of these ovarian-specific kallikrein biomarkers alone or in combination with other factors may lead to improved individualized tailoring of ovarian cancer patient therapy. Other cancers also express kallikrein proteins such as kallikrein 3 or PSA in prostate cancer and kallikreins 5, 8 and 11 in breast cancer. The kallikreins are therefore one of the most promising new cancer biomarker families and therefore deserve further study.

## Molecular markers predictive of therapeutic benefit

The predictive value of response biomarkers has been studied in an attempt to individualise patient’s treatments and for the selection of more effective therapeutic regimens. These include methylated MGMT and EGFR-TKI in glioblastoma, EGFR-TKI and ERCC1 in non-small cell lung cancer and pathologic complete response in breast cancer.

Monika Hegi, Centre Hospitalier Universitaire Vaudois (CHUV), Switzerland, updated the translational research findings from the EORTC trial 26981/NCIC CE.3. This was a randomized phase III clinical study evaluating the epigenetic inactivation of the MGMT gene as a predictive factor for benefit from adjuvant treatment with temozolomide plus radiotherapy compared with radiotherapy alone in patients with newly diagnosed glioblastoma multiforme (GBM). This study confirmed the positive predictive value of the methylated MGMT gene. At 2 years, 46% of patients who received the combined study treatment and whose tumours had a methylated MGMT promoter gene survived compared with only 14% of patients with an unmethylated MGMT gene (p=0.0001). The reproducibility of MGMT as a predictive biomarker will be prospectively validated as part of the ongoing EORTC/NCI/RTOG (EORTC protocol 26052–22053) phase III study comparing conventional adjuvant temozolomide with dose-intense temozolomide in patients with newly diagnosed glioblastoma. Numerous studies now include the assessment of MGMT status because of these findings, one of which is a phase III trial evaluating the efficacy of cilengitide in newly diagnosed GBM patients with stratification for MGMT status. Efforts are underway to identify a corresponding gene signature.

Ingo Mellinghoff (Memorial Sloan-Kettering Cancer Centre) and colleagues have studied possible biomarkers of the EGFR-TKI response in GBM. In a retrospective analysis, examining GBM tumours from patients treated with signal transduction inhibitors, such as the EGFR TKIs erlotinib or gefitinib, clinical response was highly correlated with co-expression of tumour suppressor PTEN and EGFRvIII in 10%–20% of patients. In a follow-up prospective phase I study of patients with molecularly defined PTEN deficient GBM tumours, the MTOR inhibitor rapamycin was shown to cross the blood--brain barrier. Intra-tumoural drug concentrations associated with anti-proliferative activity, similar to those observed in PTEN-deficient pre-clinical models, were documented however, no drug was identified in the tumour cells. Host-related factors (i.e. vasculature) might impede tumour cell drug delivery thereby impairing effective mTOR inhibition and the anti-proliferative response. In a subset of tumours, reactivation of Akt was associated with an inferior clinical response, leading the researchers to conclude that combination mTOR plus PI3K inhibition may overcome this type of drug resistance.

Bruce Johnson of the Dana-Farber Cancer Institute presented the impact of EGFR mutations and the potential application of EGFR-TKI treatment response biomarkers in non-small cell lung (NSCL) cancer. In NSCL, cancer EGFR mutations lie in exons 18–21 of the TK domain. A clinical study compared the relationship between EGFR gene copy number, EGFR protein expression, EGFR mutations and Akt activation status as predictive markers for gefitinib therapy in advanced NSCLC. High-EGFR gene copy number, as identified by FISH, was found to be a potential molecular predictor for gefitinib efficacy in advanced NSCLC. Two large randomised studies, one of gefitinib (Cappuzzo *et al*.) and the second, erlotinib (Tsao *et al*.) compared active treatment to placebo in patients with recurrent NSCL cancer to determine if EGFR mutation is predictive of treatment outcome. Cappuzzo concluded that high-EGFR gene copy number identified by FISH might be an effective molecular predictor for gefitinib efficacy in advanced NSCLC. However, in the Tsao *et al*. study, multivariate analysis showed that survival after treatment with erlotinib was not influenced by EGFR expression status.

Jean-Charles Soria of the Institut Gustav Roussy discussed the use of pharmacogenomic criteria, such as ERCC1 status, as a predictor of response to chemotherapy. *In vitro* and clinical data show that ERCC1 expression is negatively associated with response to cisplatin or oxaliplatin chemotherapy in gastric and colon cancer and high-tumour tissue levels of ERCC1 mRNA confers cisplatin resistance in ovarian and gastric cancer patients. Employing IHC-based ERCC1 protein expression analyses, results from the International Adjuvant Lung Cancer Trial (IALT) showed that patients with ERCC1 negative tumours randomised to adjuvant cisplatin-based chemotherapy had an increase in overall survival of 14 months compared to patients randomized to observation, whereas patients with ERCC1-positive tumours did not show this result. The Genotypic International Lung Trial (GILT) by the Spanish Lung Cancer Group conducted the first study to use ERCC1 mRNA status to customise chemotherapy in patients with previously treated NSCL cancer. Although the results suggest that high levels of ERCC1 predict a better outcome with the combination treatment of gemcitabine-docetaxel over cisplatin-docetaxel, issues of reproducibility and a trial patient drop out rate of 17% complicated the interpretation of these results. Two additional pharmacogenomic-directed trials in patients with NSCL cancer are evaluating the predictive value of ERCC1 status in relation to adjuvant cisplatin-gemcitabine (SWOG) or erlotinib with or without cisplatin (IFCT) therapy. Future trials will need to be pharmacogenomic based and integrate the best biomarkers if personalised cancer therapy is to become a reality and to explore how cisplatin resistance might be reversed by ERCC1 target modulation.

Herve Bonnefoi (Institut Bergonie) and colleagues appear to have confirmed the validity of gene expression signatures in a large series of patients with ER negative breast tumours treated in the randomised phase III EORTC 10994/BIG 00–01 neoadjuvant clinical trial. Patients received a traditional anthracycline-based (FEC) or a taxane-containing regimen (TET: docetaxel-〉epirubicin/docetaxel). Pathological complete response (pCR) defined chemosensitivity. RNA was prepared from sections of frozen biopsies taken at diagnosis and hybridised to Affymetrix X3P microarrays. A pCR was established in tumour samples from 28/66 (FEC) and 27/59 (TET) patients. *In vitro* single-agent drug sensitivity signatures were combined to obtain anthracyline- or taxane-based regimen-specific signatures and were shown to be highly predictive of pCR (p<0.0001). It appears that pCR is a good surrogate marker for ER negative tumours, and validation of these initial findings is planned.

## Oral presentation of ‘Best’ Posters

Three young European researchers presented the results of their work in the area of biomarkers and cancer therapeutics. Cathy Kelly, a Clinical Oncology Fellow, presented the initial findings of research done in the area of breast cancer progression-associated biomarkers at the UCD School of Biomolecular and Biomedical Science, Conway Institute, University College Dublin, Ireland. This group selected 137 targets for antibody production from a cohort of several hundred candidate progression-associated biomarkers by applying a novel bioinformatic technique to available DNA microarray datasets. The first 32 antibodies produced were screened via Western blot and IHC against normal and tumour tissue as well as cultured cells represented on tissue microarrays (TMAs). The highly optimized antibodies were screened against a TMA constructed from a cohort of 524 cases of breast cancer that included all disease stages. Differential staining between normal and breast cancer tissue was observed in a quarter of the 32 antibodies. PDZK1, an oestrogen-responsive gene associated with good prognosis showed differential expression between ER positive and negative tumour cell lines, confirmed by IHC and PDZK1 protein expression was associated with improved breast-cancer-specific survival, ER positivity and low-tumour grade. The researchers concluded that re-analysis of publicly available DNA microarray datasets is a cost-effective method for candidate biomarker selection/prioritization, and good quality TMAs can be used to validate both prognostic and predictive biomarkers.

Sabine Tejpar and colleagues at the Catholic University of Leuven, Belgium, have identified a potential biomarker of response to cetuximab therapy in colorectal cancer (CRC). Recent reports have shown that metastases from patients responding to cetuximab monotherapy show high mRNA expression of amphiregulin (AREG) and epriregulin (EREG). This group examined AREG and EREG expression by real-time quantitative RT-PCR (TaqMan) in FFPE tumour primary CRC tissue from patients with irinotecan-resistant metastatic CRC treated with cetuximab and irinotecan and correlated expression with response and survival. The results confirm that high AREG and EREG levels in CRC tissue from the primary site were associated with objective response and improved overall survival, and further validation of these markers is warranted.

Marlies Langenberg, Department Medical Oncology, Utrecht Medical Center in the Netherlands presented the results of her group’s evaluation of circulating endothelial progenitor cells (CEPC) as biomarkers. CEPC are known to reflect tumour angiogenic activity and are therefore of potential use as biomarkers in the clinical decision-making process. However, CEPC have yet to be validated for routine use due to differences in the technical procedures employed for measuring CEPC and the significant inter-patient and intra-patient variability observed. To address these issues, CEPC were measured in over 1000 samples from 450 clinical trial patients treated with various modalities and 90 volunteers. Intra-procedure, intra-patient and inter-patient variability were analysed. The results show that flow cytometry is a reliable and preferred method of CEPC measurement due to its low intra-procedure and intra-patient variability and because it enables specific phenotypic characterization of cells in stored samples. However, although absolute cell number is reflective of biological activity, significant inter-patient variability was observed, thus prohibiting the use of reference values. These findings help to understand the technical issues surrounding the use of circulating endothelial progenitor cells as biomarkers, and the conclusions are consistent with the most recent ASCO recommendations, concerning the use of circulating tumour cells as clinical tumour markers.

## Conclusions

This tripartite annual meeting, once again, provided participants with a broad overview of the current state of cancer biomarker research, the clinical application of specific markers in all major tumour types and helped to advance and provide direction for future biomarker research, thus ensuring continued international scientific collaboration between Europe and the United States. Planning is already underway for the 2008 ASCO-NCI-EORTC Annual Meeting on ‘Molecular Markers in Cancer’ to be held in Hollywood, FL, USA, on 30 October–1 November 2008.

